# Comparison study of exhaust plume impingement effects of small mono- and bipropellant thrusters using parallelized DSMC method

**DOI:** 10.1371/journal.pone.0179351

**Published:** 2017-06-21

**Authors:** Kyun Ho Lee

**Affiliations:** Department of Aerospace Engineering, Sejong University, Seoul, Republic of Korea; North China Electric Power University, CHINA

## Abstract

A space propulsion system is important for the normal mission operations of a spacecraft by adjusting its attitude and maneuver. Generally, a mono- and a bipropellant thruster have been mainly used for low thrust liquid rocket engines. But as the plume gas expelled from these small thrusters diffuses freely in a vacuum space along all directions, unwanted effects due to the plume collision onto the spacecraft surfaces can dramatically cause a deterioration of the function and performance of a spacecraft. Thus, aim of the present study is to investigate and compare the major differences of the plume gas impingement effects quantitatively between the small mono- and bipropellant thrusters using the computational fluid dynamics (CFD). For an efficiency of the numerical calculations, the whole calculation domain is divided into two different flow regimes depending on the flow characteristics, and then Navier-Stokes equations and parallelized Direct Simulation Monte Carlo (DSMC) method are adopted for each flow regime. From the present analysis, thermal and mass influences of the plume gas impingements on the spacecraft were analyzed for the mono- and the bipropellant thrusters. As a result, it is concluded that a careful understanding on the plume impingement effects depending on the chemical characteristics of different propellants are necessary for the efficient design of the spacecraft.

## Introduction

**Fig 1:**
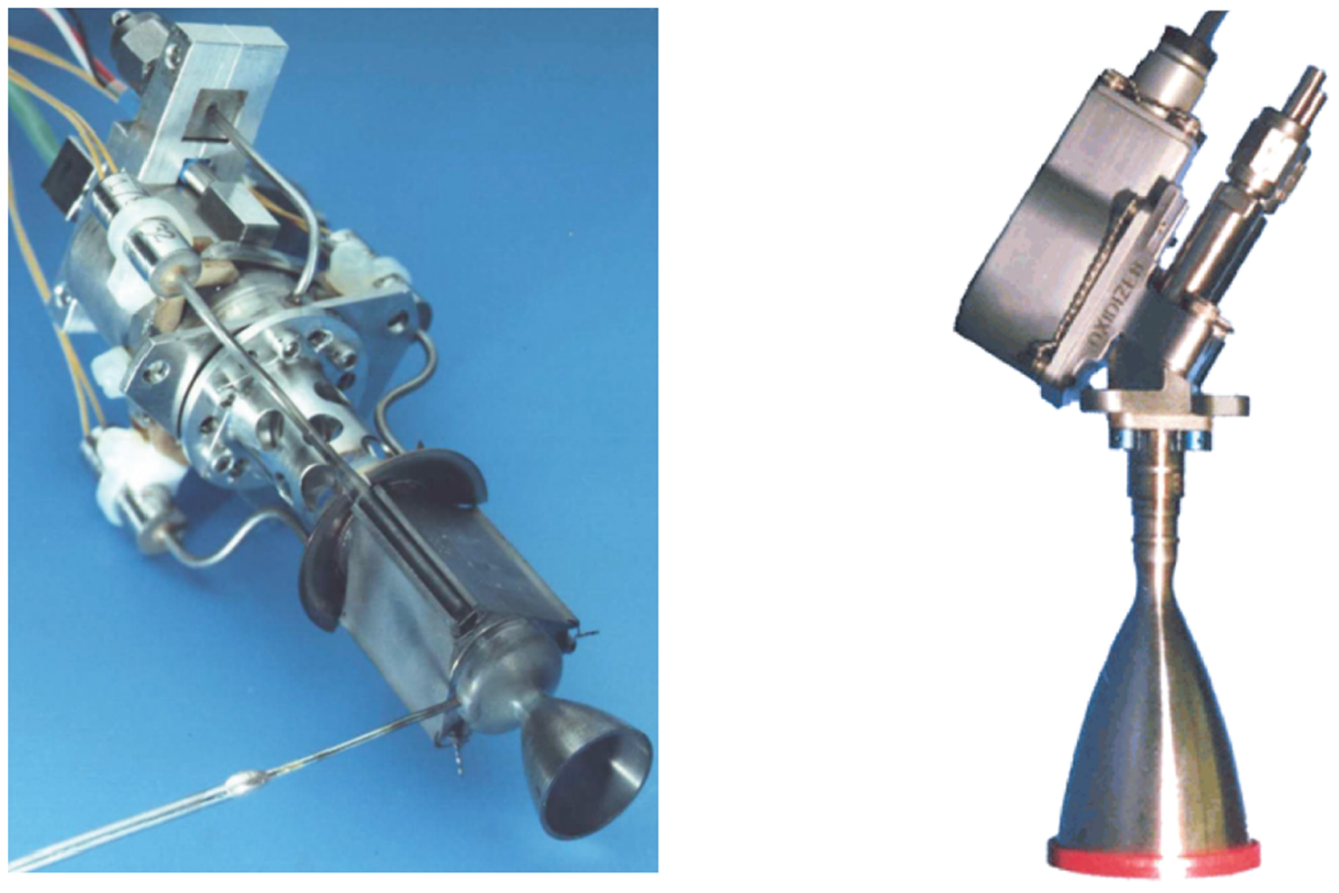
Examples of small mono- and bipropellant thrusters. (A) Monopropellant thruster (Hydrazine propellant) [1]. (B) Bipropellant thruster (MMH-NTO propellant) [1].

**Fig 2:**
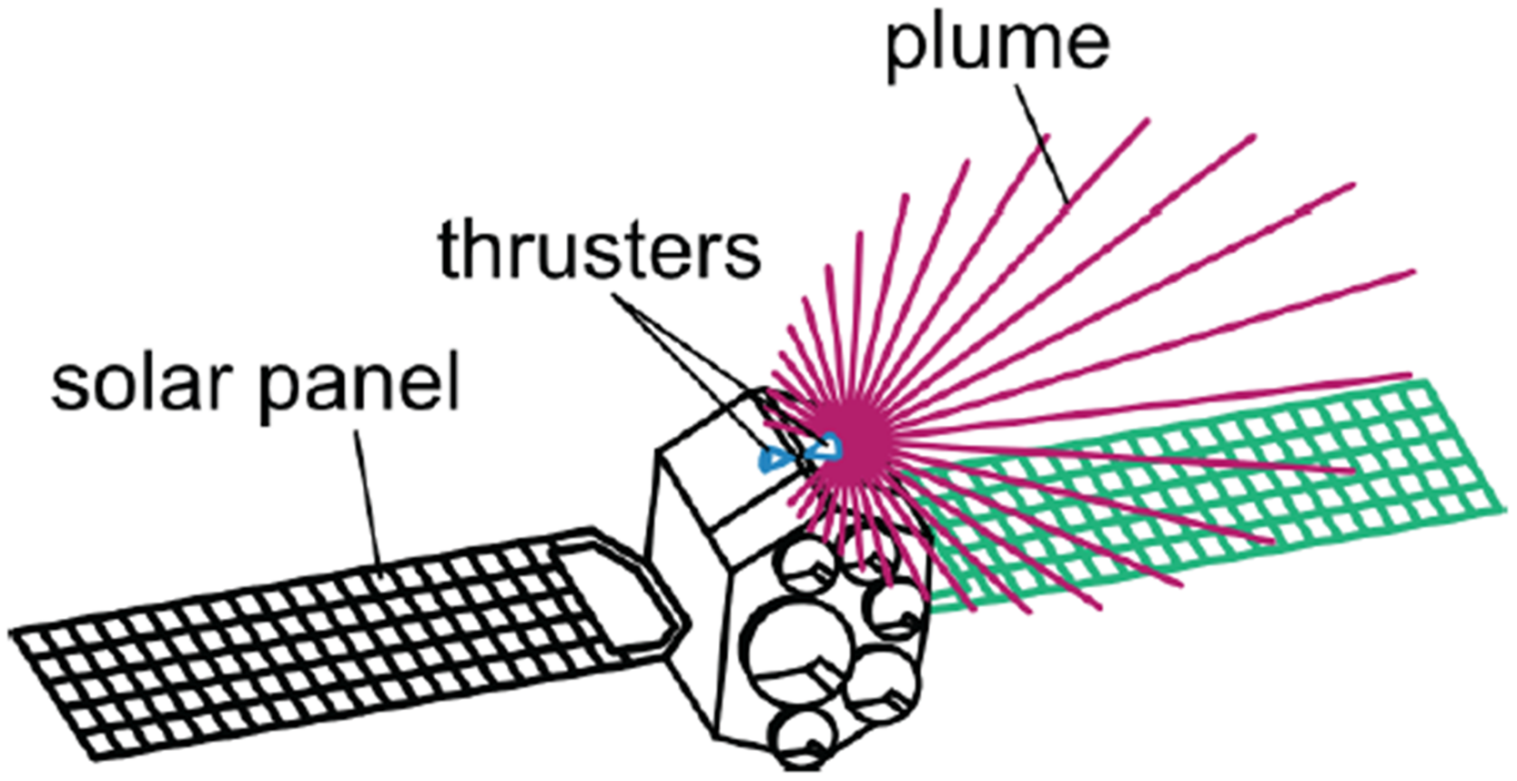
Plume impingement concept of small thruster [2].

Generally, a space propulsion system generates a reaction force (thrust) to perform normal mission operations of a spacecraft through the ejection of a specific amount of a high speed propellant gas flow into a vacuum space environment. Among the various system types, a low thrust liquid rocket engine, or a small thruster, provides a precise thrust or/and impulse bit for attitude control, drag make-up and orbit transfer maneuvers of the spacecraft by generating the kinetic energy of the propellant gas flow through a nozzle expansion from the chemical reaction energy of the liquid propellants in the combustion chamber. This exhaust combustion gas flow in high-temperature and high-pressure is defined as the plume gas flow. Depending on the characteristics of the liquid propellants, the low thrust liquid rocket engines can be divided into two main types as seen in Fig 1 [1]: a monopropellant thruster which needs one single fuel which decomposes into hot gas when in contact with the solid catalyst, and a bipropellant thruster which requires a fuel and oxidizer separately to generate a high-pressure hot combustion gas [1]. As the exhaust plume gas flow expands freely in a vacuum space environment along all directions and collides with spacecraft surfaces directly shown in Fig 2 [2], the plume gas impingement can cause adverse influences during mission operations of the spacecraft, such as a disturbing force/torque, severe thermal loading, and critical contamination of sensitive components and sensors [2]. Since several failures of the function and performance of the spacecraft due to the plume effects have been reported, an accurate assessment and a minimization of undesirable plume impingement effects are a requisite verification process for the spacecraft design when using the low thrust liquid rocket engines [2]. For an efficient prediction, various numerical methods have been developed recently to simulate the physical characteristics of the plume gas flowfields rather than experimental approaches [2]. Among such numerical methods, the Direct Simulation Monte Carlo (DSMC) method [3,4] has been majorly used to analyze the plume gas flow because it can predict an accurate flowfields in a rarefied transition and a free molecular regime under a vacuum environment space by solving the Boltzmann equation statistically. Thus, various studies have investigated the exhaust plume gas flow behaviors of the small thrusters using the DSMC method [5–16].

However, the mono- and bipropellant thrusters have been employed as the representative space propulsion systems for the various spacecraft, quantitatively comparison studies of the exhaust plume flow impingement effects of small mono- and bipropellant thrusters have not been investigated yet based on a literature review. Actually, major selection criteria of the propellant and its operating system have been usually focused on the performance parameters such as a thrust level and a specific impulse, etc. rather than the exhaust plume effects. But as the plume gas flows generated from these two propellants have different characteristics in terms of chemical, thermal and fluid properties, it would be anticipated that their collisions consequently cause different impingement effects on the spacecraft. Because the evaluation of the plume gas flow influences is one of the essential design verification tasks, understanding fundamental characteristics of the overall plume impingement effects depending on the different propellant types can provide useful information for reducing a development cost and time at the initial design phase.

Thus, the aim of the present study is to investigate and compare the major differences of the plume gas flow impingement effects quantitatively between small mono- and bipropellant thrusters using the computational fluid dynamics (CFD). For an efficiency of the numerical calculations, the whole calculation domain is divided into two different flow regimes depending on the flow characteristics, and then Navier-Stokes equations and parallelized Direct Simulation Monte Carlo (DSMC) method are adopted for each flow regime sequentially so that individually calculated results could be combined as boundary conditions for other methods. Through the present analysis results, thermal and mass influences of the plume gas impingements on the spacecraft including number flux and heat flux were investigated between the mono- and the bipropellant thrusters. As a result, it is anticipated that the present study could provide practically useful information to the related engineers on determining the proper propulsion system type and evaluating the spacecraft system design through investigations of the plume impingement effects of the mono- and bipropellant thrusters.

## Numerical methodology

### Parallel Direct Simulation Monte Carlo method

The flow regimes can be generally characterized by the Knudsen number (*Kn*), which depicts a comparison between an actual characteristic length and a mean free path of gas particles. When the Knudsen number of a specific flow is greater than a unity, the mean free path of flow molecules can be comparable to an actual length of the physical problem. Therefore, a flow model based on a continuum assumption such as the N-S equation cannot be available for a good approximation any more in case of the high Knudsen number flow. Thus, a kind of molecular dynamic simulations with statistical methods should be employed for the rarefied flow regime to deal with the Boltzmann equation in a nonlinear form of Eq (1) [3,4]

$$\frac{\partial }{\partial t}\left(nf\right)+\vec{v}\frac{\partial }{\partial \vec{r}}\left(nf\right)+\vec{F}\cdot \frac{\partial }{\partial \vec{v}}\left(nf\right)=\int_{-\infty }^{\infty }\int_{0}^{4\pi }n^{2}\left(f^{*}f_{1}^{*}-ff_{1}\right)v_{r}\sigma d\Omega \;d\vec{v_{1}}$$ (1)

In Eq (1), *n*, $\vec{v}$, $\vec{r}$, *f* and *v*_*r*_ are number density, velocity vector, position vector, probability density function and relative velocity of molecules, respectively. Also, $\vec{F}$, $d\vec{v_{1}}$, d*Ω* and *σ* are external force vector, molecules of class with velocity *v*_1_, elementary solid angle and collision cross section [3]. Among various molecular dynamic simulation methods, the Direct Simulation Monte Carlo (DSMC) method proposed by Bird [3] is regarded as the most effective technique for solving the Boltzmann equation to deal with the rarefied flow. The DSMC is a statistical particle simulation method based on kinetic theory, which uses the representative particles to trace in space and time to simulate the physical behaviors of the real gas [3]. But the DSMC method has some drawbacks because it should evaluate numerous intermolecular interactions between all the simulated particles. For example, it is generally found that the DSMC method required a much longer computational time than the conventional continuum flow models because it considered all the interactions of every simulated molecules sequentially. As the present study intends to analyze the plume impinging influences on a complex three dimensional unstructured grids of the actual satellite configuration, a parallelized DSMC code was employed to increase computational efficiencies such as a faster calculation time with 28 CPU cores. The variable hard sphere (VHS) model [3] is used as the intermolecular-collision model and the no-time counter (NTC) method is for the collision sampling technique [3]. The Larsen-Borgnakke model [17] is employed to redistribute the translational and the internal energy exchange between the gas molecules. In addition, the unstructured three dimensional grid system of the computation domain was modeled using commercial software, GRIDGEN that adopts the Delaunay technique [18]. The parallel processing was made by dividing the computational domain into several subdomains by using the MeTiS library, which is based on the k-way, n-partitioning technique by Karypis [19]. After each time step of Δt = 1×10^−7^ sec, information about the particles and their properties was exchanged through the subdomain boundary by using the Message Passing Interface (MPI) library [20]. Also, flow properties were averaged over a large number of sampling steps about 10,000 to minimize statistical scatter. A steady state was typically completed after 500 transient steps, and additional 5,000 sampling steps were conducted to obtain the time averaged flow properties.

## Results and discussion

For efficiency and accuracy of the numerical calculations, the calculation domain was composed of two different flow regimes depending on the flow characteristics, which were a 2-D axisymmetric continuum flow domain inside the nozzle and a 3-D rarefied plume gas flow domain under the vacuum environment, respectively. By doing this, appropriate numerical methods could be combined and applied to each subdomain sequentially to use numerical solutions obtained from one method as the boundary conditions for others.

### Inlet and boundary conditions of plume simulation

**Fig 3:**
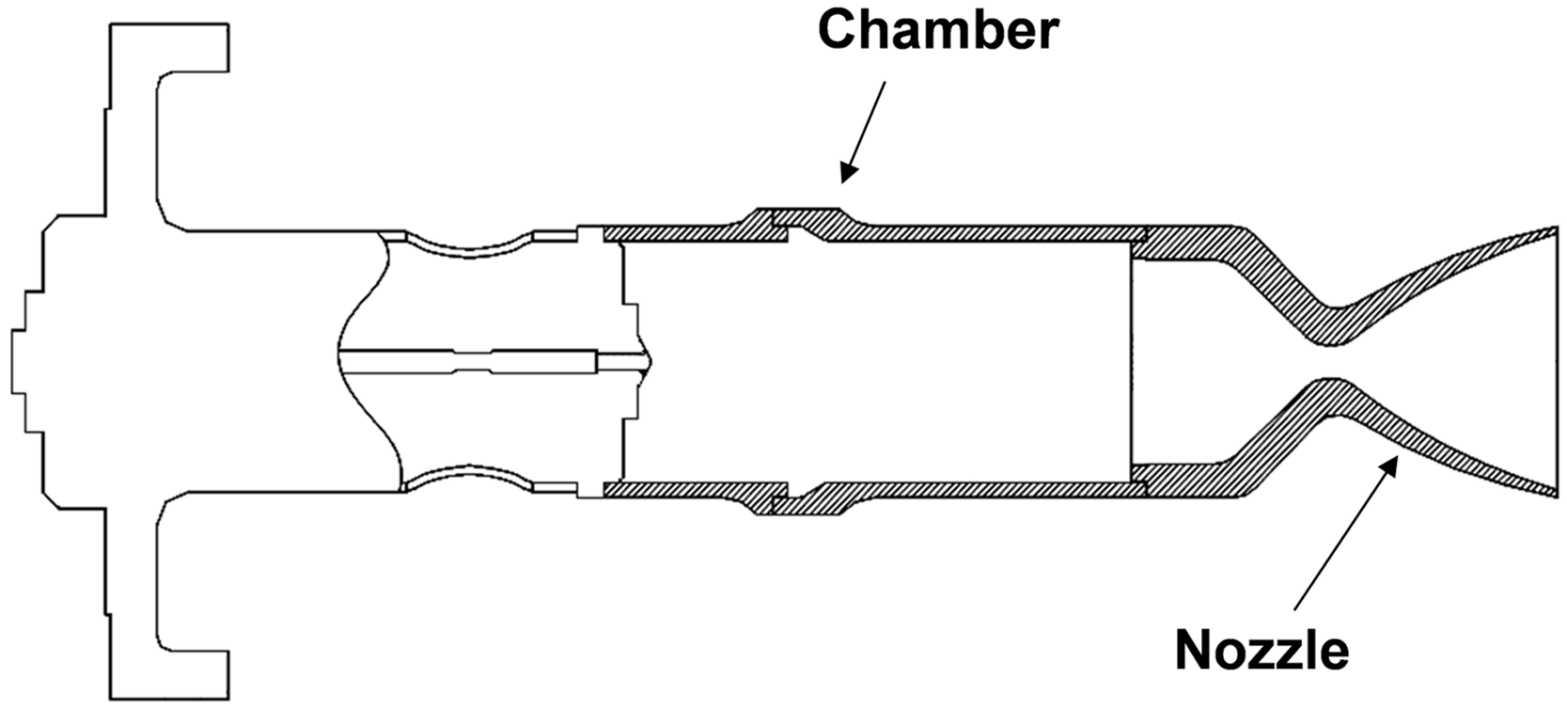
Small thruster configuration [10,16].

As the plume gas behaviors in the vacuum are dominantly affected by the continuum nozzle flow inside the thruster, an accurate estimation of the plume flowfields was required to define the inlet boundary condition at the nozzle exit plane for the plume analysis. Thus, the N–S equations were solved numerically to predict continuum gas flow distributions inside the thruster nozzle. The small thruster configuration considered in this study is illustrated in Fig 3. It has a conical nozzle with an expansion ratio of 50:1 and it assumed to be provide a five newton reaction force at the stagnated chamber pressure (*p*_*c*_) = 1.45 MPa [16].

For the boundary conditions, the stagnation conditions inside the chamber, such as mole fractions of the combustion gas species, molecular masses of gas mixture, and adiabatic flame temperatures, were calculated as given in Table 1 by solving the chemical equilibrium reaction equations and then they were specified as an inlet condition of the nozzle [16]. Also, ten kinds of product gas species in Table 1 were regarded as a mixture of perfect gases in Eq (2), and their compositions were treated as a chemically frozen flow during the nozzle expansion process [16].

$$p=\rho RT\overset{N}{\underset{i=1}{\sum }}\frac{Y_{i}}{M_{i}}$$ (2)

Here, *Y*_*i*_ and *M*_*i*_ are the mass fraction and the molecular mass [g/mol] of the product gases, and *R* is the universal gas constant (8314.41 J/kmol K), respectively.

**Table 1:** Chemical equilibrium reaction results of hydrazine and MMH-NTO [1,6].

Results	Hydrazine	MMH-NTO
Mole fractions of combustion gas species		
*H*_*2*_	0.35761	0.15657
*N*_*2*_	0.27152	0.30513
*NH*_*3*_	0.37087	-
*H*_*2*_*O*	-	0.32741
*CO*	-	0.13145
*CO*_*2*_	-	0.03628
*H*	-	0.02133
*NO*	-	0.00235
*O*	-	0.00131
*OH*	-	0.01709
*O*_*2*_	-	0.00108
Molecular mass of gas mixture [g/mol]	14.62	20.46
Adiabatic flame temperature [K]	1342.8	3087.4

**Fig 4:**
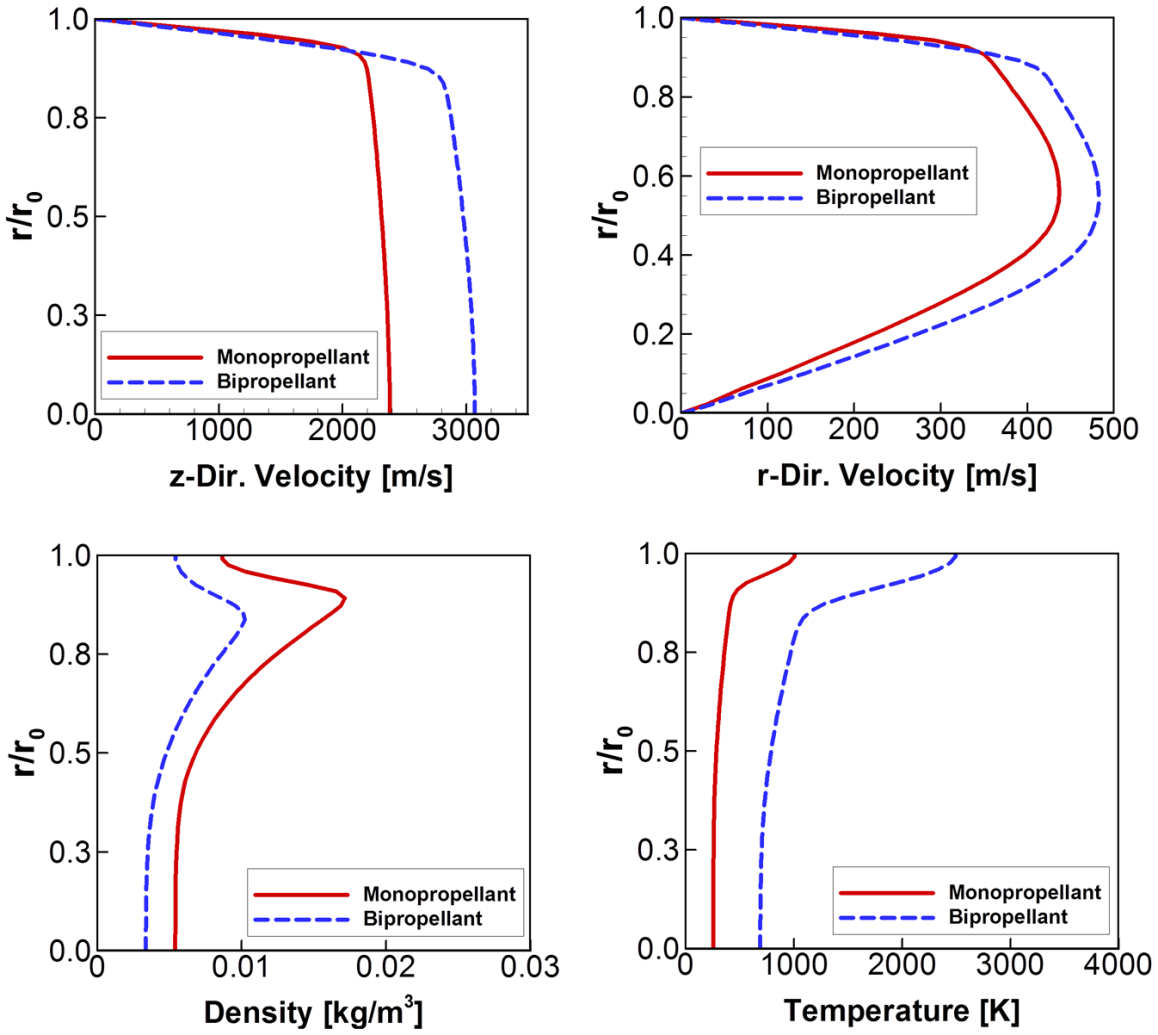
Exhaust plume flowfields at the thruster nozzle exit plane by N-S equations [16].

As a result, the propellant gas flowfields at the nozzle exit plane can be plotted as Fig 4 including the two velocity components, density, and temperature [16]. First, the temperature of the bipropellant gas is observed much higher than that of the monopropellant because the adiabatic temperature of MMH-NTO reaches about 3,087 K in the chamber while the decomposition temperature of the hydrazine is only about 1,343 K as seen in Table 1. For example, the gas temperatures at the center of the nozzle exit plane were estimated roughly as 260 K for hydrazine and 690 K for MMH-NTO propellant, respectively. As a result, higher velocities were also estimated for the MMH-NTO thruster because the exhaust velocity tends to increase proportional to the chamber temperature based on the general rocket performance equation. However, a gas flow with a higher density was produced for the hydrazine decomposition opposite to the temperature distribution. The reason is that the internal nozzle flow was assumed to obey the perfect gas law in Eq (2), which is defined as an inverse relation between the density and the temperature at a given pressure. Moreover, some drastic variations of the flowfields were found in the given profiles nearby the nozzle wall due to the boundary layer effect.

For the three dimensional DSMC simulation of the present study, the nozzle exit plane is chosen as the breakdown face between the continuum flow and the rarefied flow domains, and this assumption is suitable for most nozzles when the exit-to-throat area ratio are not very large [9]. Then the continuum flowfield results at the nozzle exit in Fig 4, which were obtained by the N-S equations, were applied directly as the inflow boundary condition for the DSMC method together with the gas mixture compositions given in Table 1 using the Maxwell distribution function based on the previous researches [5–14]. In the present study, a steady state flow is considered and the plume flow is assumed as a mixture of single-phase ideal gases without any solid/liquid particulates. Also, a nozzle outside space is assumed as a vacuum condition while the gas pressures at the nozzle exit plane is much higher, which means there is no inverse flux of a free stream into the nozzle inside through the nozzle exit plane. And most of the exhaust flow at the nozzle exit plane is a supersonic condition while a subsonic flow region is very limited. As a result, one-way coupling approach is employed between the N-S equations and the DSMC interface based on the previous researches [5–14].

For the three dimensional DSMC simulation of the present study, the continuum flowfield results at the nozzle exit in Fig 4 were applied directly as inlet conditions together with the gas mixture compositions given in Table 1. In the case of the solid boundaries, the satellite structure and components were all modeled as a diffusely reflected surface with complete energy accommodation. Additionally, the coldest surface temperatures of each component and the structure during satellite operation listed in Table 2, which were obtained from an orbit simulation of the satellite, were used as a solid wall boundary temperature in the present DSMC analysis to estimate heat fluxes on the satellite surfaces and components caused by the impinging plume particles. The remaining boundaries were assumed to be a particle sink to simulate a vacuum condition.

**Table 2:** Coldest Temperature of satellite components and structure.

Satellite components	Temperature [K]
Thruster	158.0
Solar panel	200.0
POD antenna	155.0
S-band antenna	155.0
Bottom platform	142.0
Launch vehicle adapter ring	151.0

### Plume flow impingement effects on spacecraft

**Fig 5:**
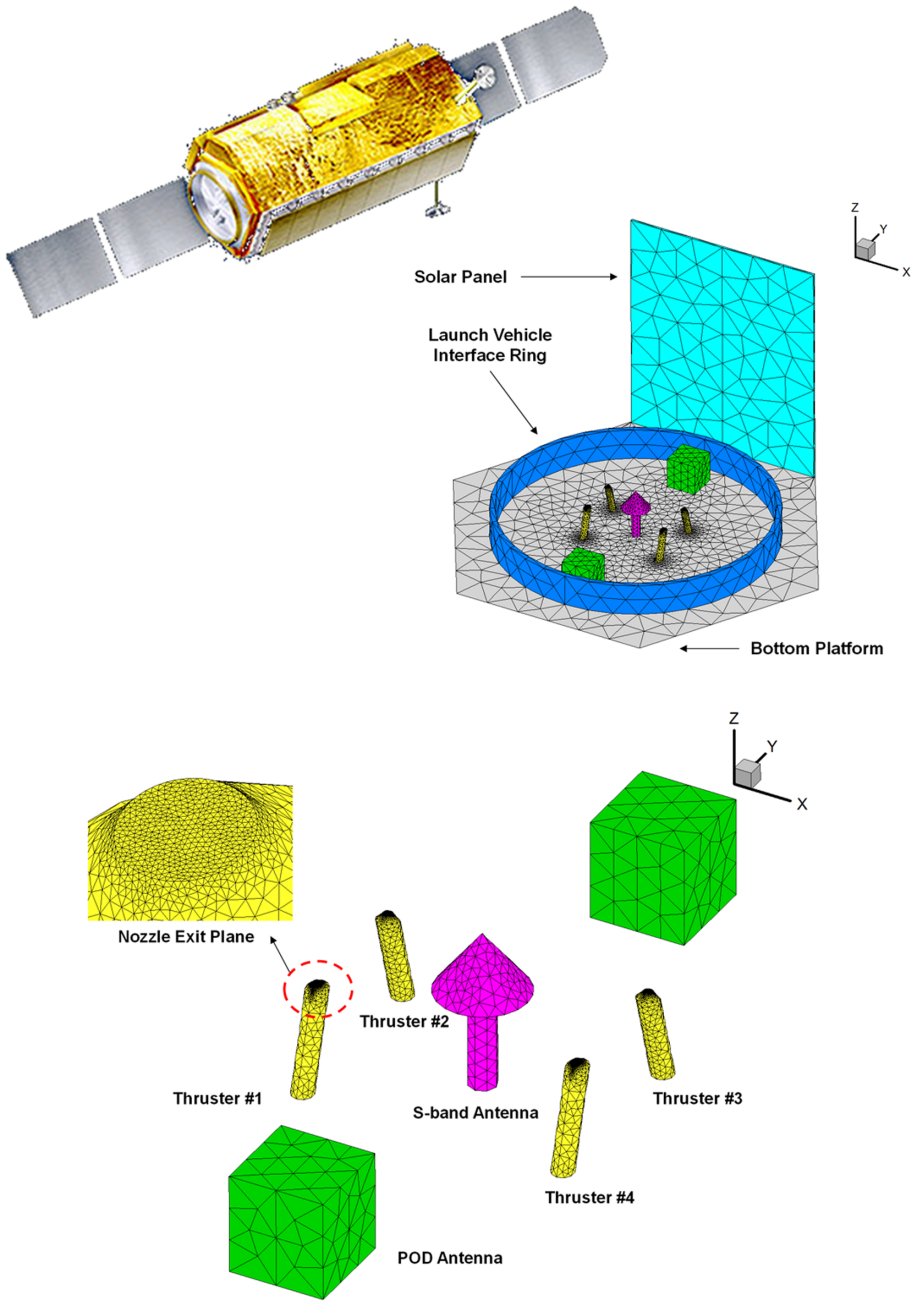
Calculation domain of plume impingement for DSMC method. (A) Example of the satellite configuration. (B) Computational grid with satellite bottom platform. (C) Detailed grid inside bottom platform.

The overall configuration of the satellite considered in the present study is shown in Fig 5A. A large sized solar panel was installed onto the side of the satellite following a longitudinal axis direction to generate higher electrical power from the sun. A numerical grid system for the bottom platform of the satellite and the major components is given in Fig 5B. One S-band antenna and four reaction control thrusters were located on the bottom platform, which were surrounded by a launch vehicle interface ring. Additionally, two antenna modules for a precision orbit determination (POD) were modeled simply adjacent to the thrusters. Part of the solar panel was attached vertically to the bottom platform of the satellite outside the ring with a 1 m height. The nozzle exit plane of each thruster was canted outward to the vacuum space to provide the necessary thrust and momentum for efficient attitude control as shown in Fig 5C. The three-dimensional unstructured geometry was generated with the GRIDGEN commercial software [18] using about 24,000 nodes and 120,000 tetrahedron cells. The cell size at the vicinity of the thruster exit was generated sufficiently smaller than the local mean free path to guarantee good accuracy. To simulate the vacuum boundary condition, the computational domain was extended to 3.2 m in radius and 2.0 m in height from the center of the bottom platform of the satellite. Because the height of the solar panel is higher than the interface ring and two kinds of antennas were located beside the thrusters, all the components including the satellite structures have a strong possibility of being exposed directly to the exhausted plume gas flow. To reflect the maximum plume impingement effects, the present study considered a simultaneous firing condition of all four thrusters as a representative case.

**Fig 6:**
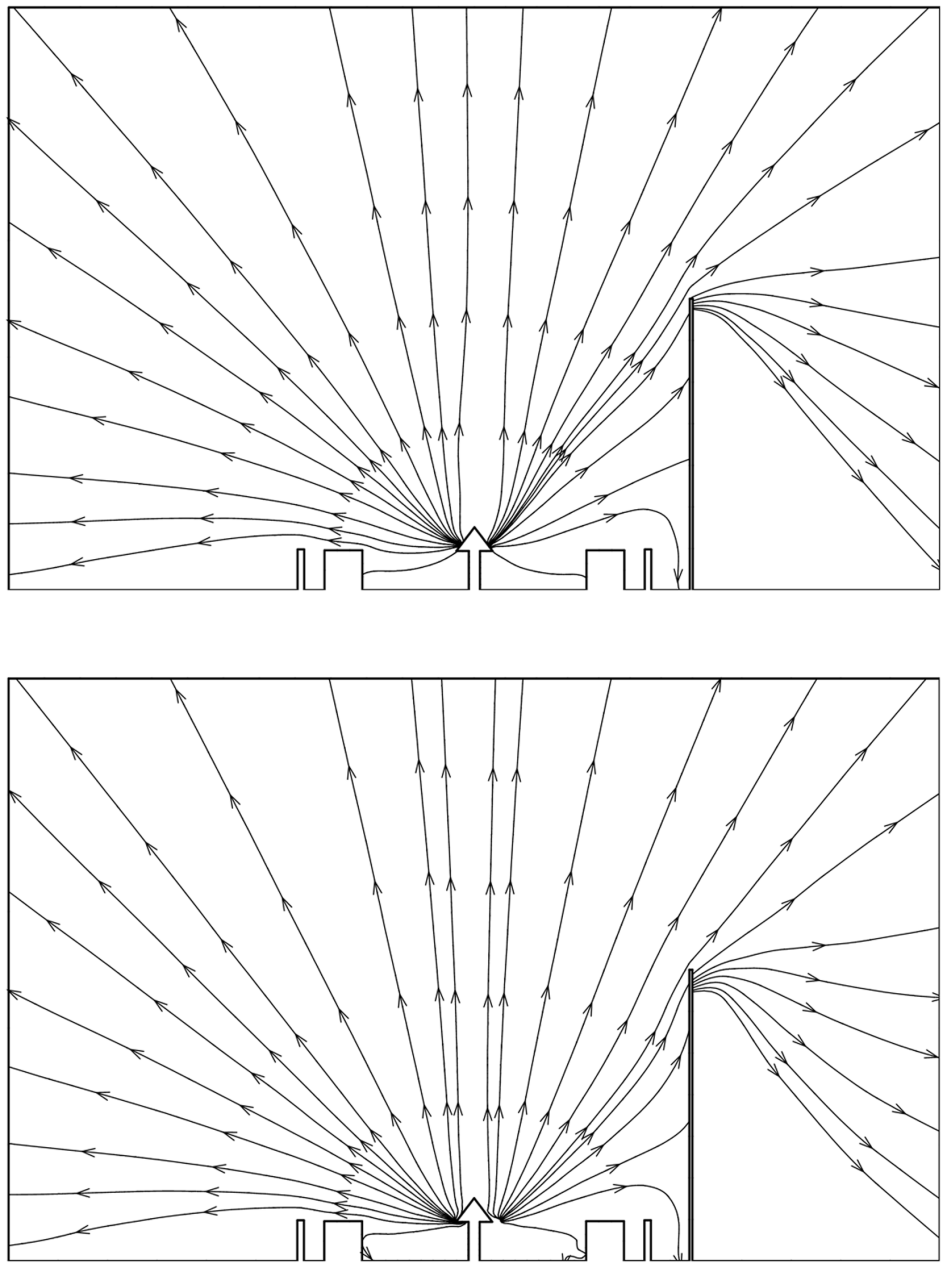
Velocity streamlines of plume gas flow. (A) Monopropellant hydrazine. (B) Bipropellant MMH-NTO.

As a first outcome, the overall plume flow behaviors in the vacuum space, such as the velocity streamline, number density and overall temperature, were compared for both propellants. The velocity streamlines of the mono- and bipropellant plume gases at the center cross section of the platform are shown in Fig 6. Both hydrazine and MMH-NTO plume gases expanded with similar patterns as the simulated plume particles, which were initially injected from the four nozzle exits of the firing thrusters, spread primarily outward to the vacuum space far from the satellite. Because the plume particles expanded simultaneously, some portion of them merged together and formed a main flow stream at the center of the bottom platform for both propellants. Then, a considerable amount of the plume particles directly collided with the solar panel because a large portion of the panel area was exposed directly to a main stream of the exhaust plume flow. Thus, undesirable plume effects including disturbance force/torque, heat load, and chemical species deposition could become critical at the solar panel due to severe plume gas impingement. Moreover, it was predicted that a small amount of plume backflow, which was directed to the POD and S-band antenna components inside the interface ring, could cause additional impingement effects.

**Fig 7:**
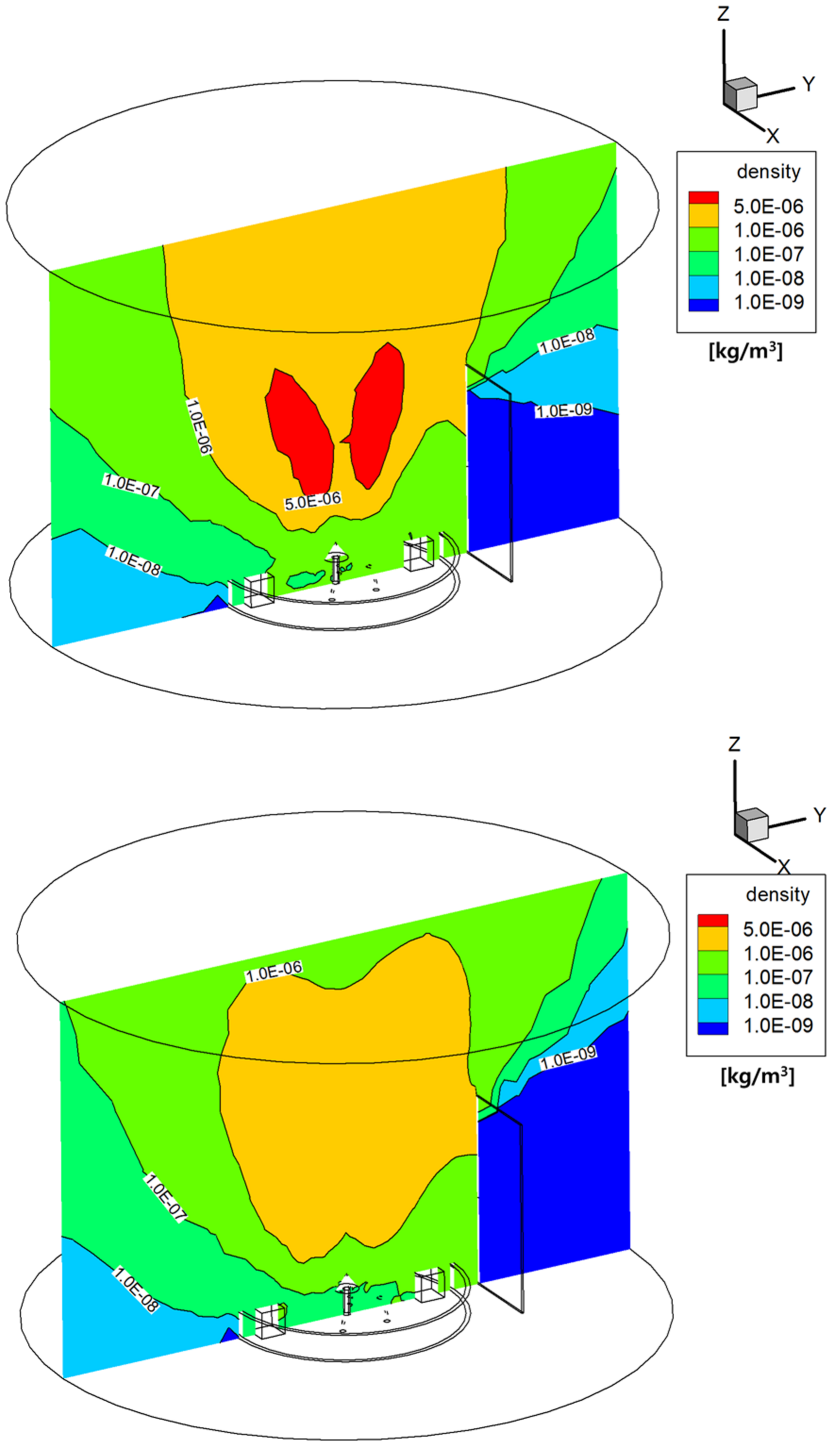
Density distributions of plume gas flow [kg/m^3^]. (A) Monopropellant hydrazine. (B) Bipropellant MMH-NTO.

**Fig 8:**
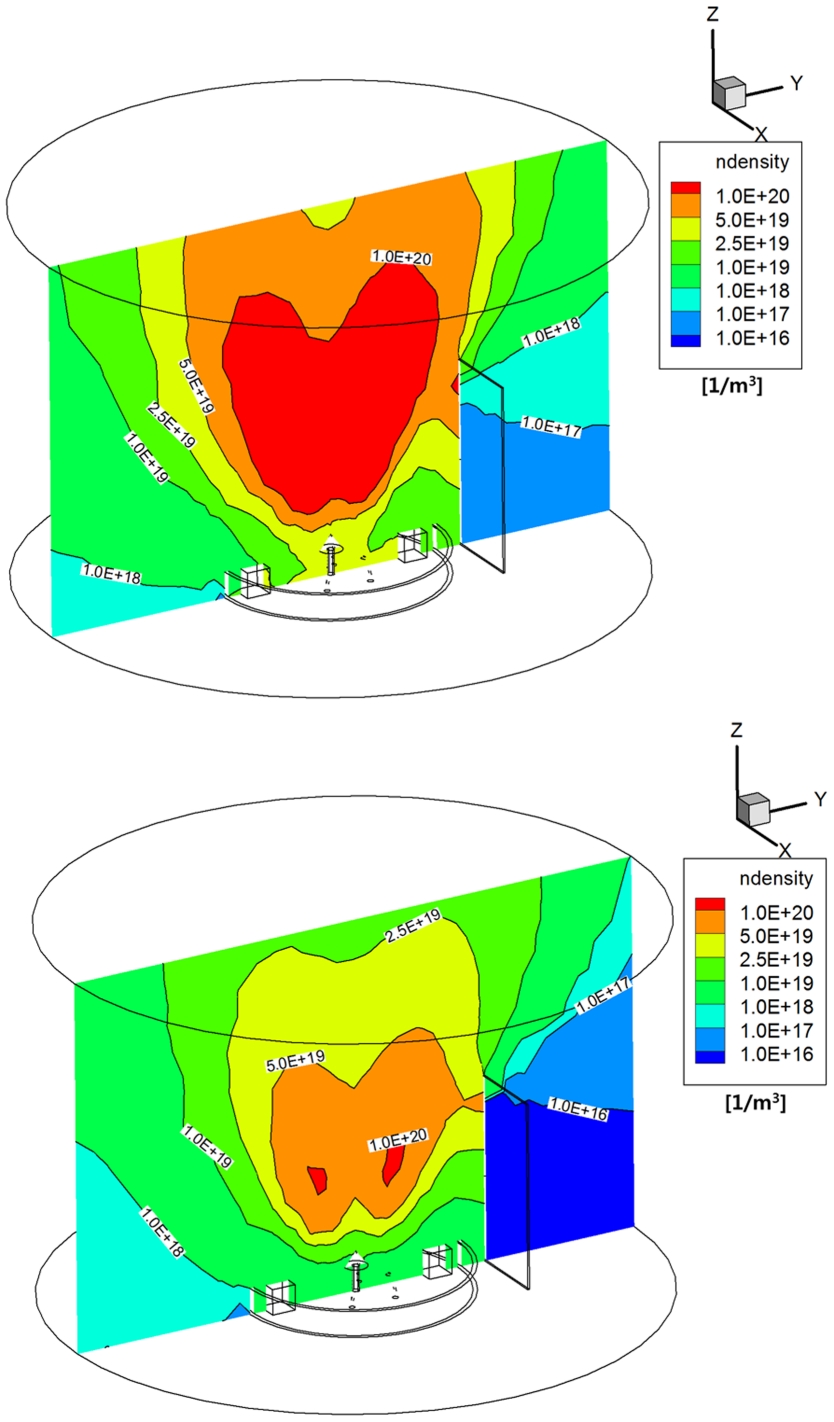
Number density distributions of plume gas flow [1/m^3^]. (A) Monopropellant hydrazine. (B) Bipropellant MMH-NTO.

Regarding to the density distribution of the plume gas, Fig 7A shows that the monopropellant hydrazine plume gas flow spread more densely all over the calculation domain including the backflow regions inside the interface ring and outside the solar panel than that of the bipropellant MMH-NTO gas shown in Fig 7B because a higher density profile was initially applied at the inflow condition of the DSMC method based on the continuum flow results inside the thruster nozzle. This can be confirmed more clearly when converted into the number density distributions shown in Fig 8. It was estimated that the number density of the hydrazine plume particles ranged roughly between 1.0E+18 1/m^3^ and 3.0E+19 1/m^3^ near the antenna components and inside the interface ring shown in Fig 8A, while it was less than 1.0E+19 1/m^3^ for the MMH-NTO plume gas from Fig 8B. Additionally, the order of the number density at the core region of the main plume stream was over 1.0E+20 1/m^3^ for the hydrazine gas and 5.0E+19 1/m^3^ for the MMH-NTO gas, respectively. As a result, a larger number of hydrazine plume particles were found to be distributed in the proximity of the solar panel and in the backflow regions inside the interface ring. Thus, it was anticipated that the high number density of the hydrazine plume particles could cause a significant increase in the collision possibility of the plume particles onto the spacecraft components and structure including the solar panel rather compared to the MMH-NTO plume gas.

**Fig 9:**
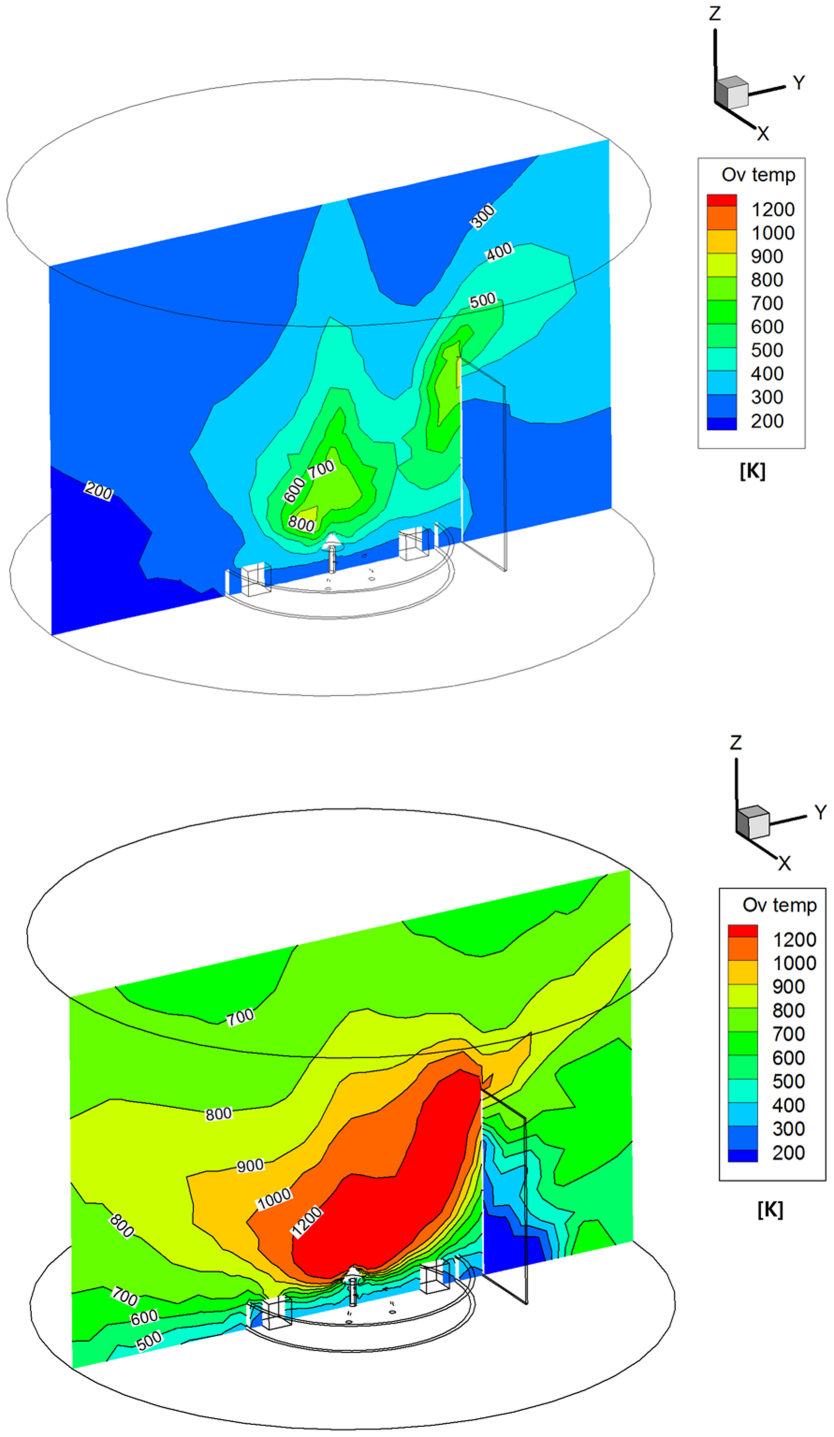
Overall temperature distributions of plume gas flow [K]. (A) Monopropellant hydrazine. (B) Bipropellant MMH-NTO.

In addition, the difference in the overall temperatures between the hydrazine and MMH-NTO plume flow gases were compared in Fig 9. In contrast to the number density, a higher temperature plume gas for the MMH-NTO propellant was observed to be distributed over the whole calculation domain according to the continuum flow temperature profile at the nozzle exit plane shown in Fig 4. The MMH-NTO plume particles ranged between 600 K ~ 1,000 K around the antenna components inside the interface ring from Fig 9B, whereas lower temperatures were calculated for the hydrazine propellant that ranged between 200 K ~ 500 K as seen in Fig 9A. Moreover, high temperature regions were observed in the vicinity of the upper portion of the solar panel because a considerable amount of thermal energy from the plume gas molecules was converted from their reduced kinetic energy which was proportional to the increased collision of the plume particles on the surface of the solar panel. Thus, the plume gas temperature varied gradually from 300 K to 800 K for the hydrazine and from 400 K to 1,200 K for the MMH-NTO following the height of the solar panel. These results indicate that the plume gas temperature was greatly dependent on the amount of thermal energy released from the chemical reactions of the propellant. Thus, it was anticipated that plume gas impingement could exert a severe thermal loading influence on the spacecraft components and structure depending on the type of propellant used.

**Fig 10:**
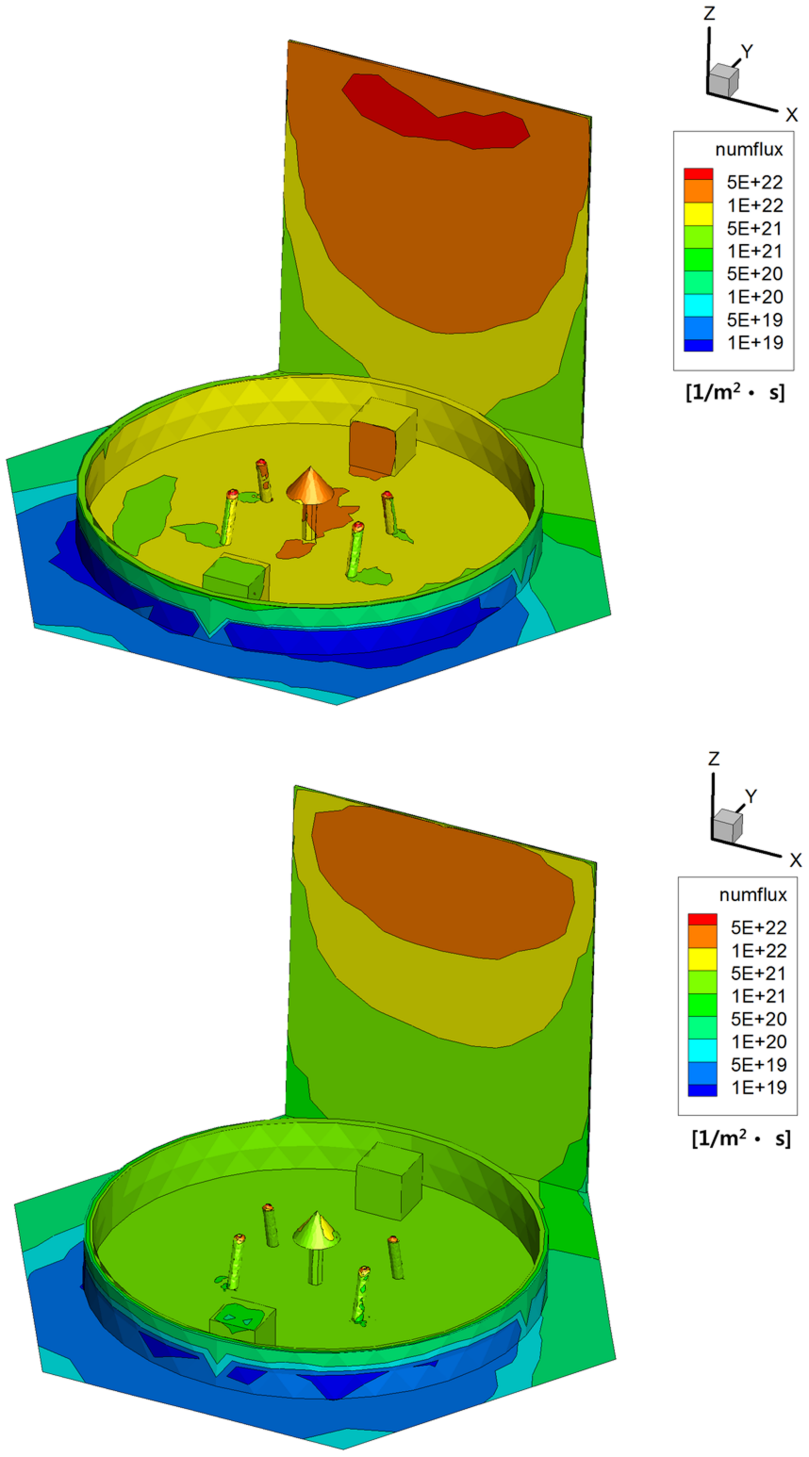
Surface number flux distributions of plume gas flow [1/m^2^·s]. (A) Monopropellant hydrazine. (B) Bipropellant MMH-NTO.

**Fig 11:**
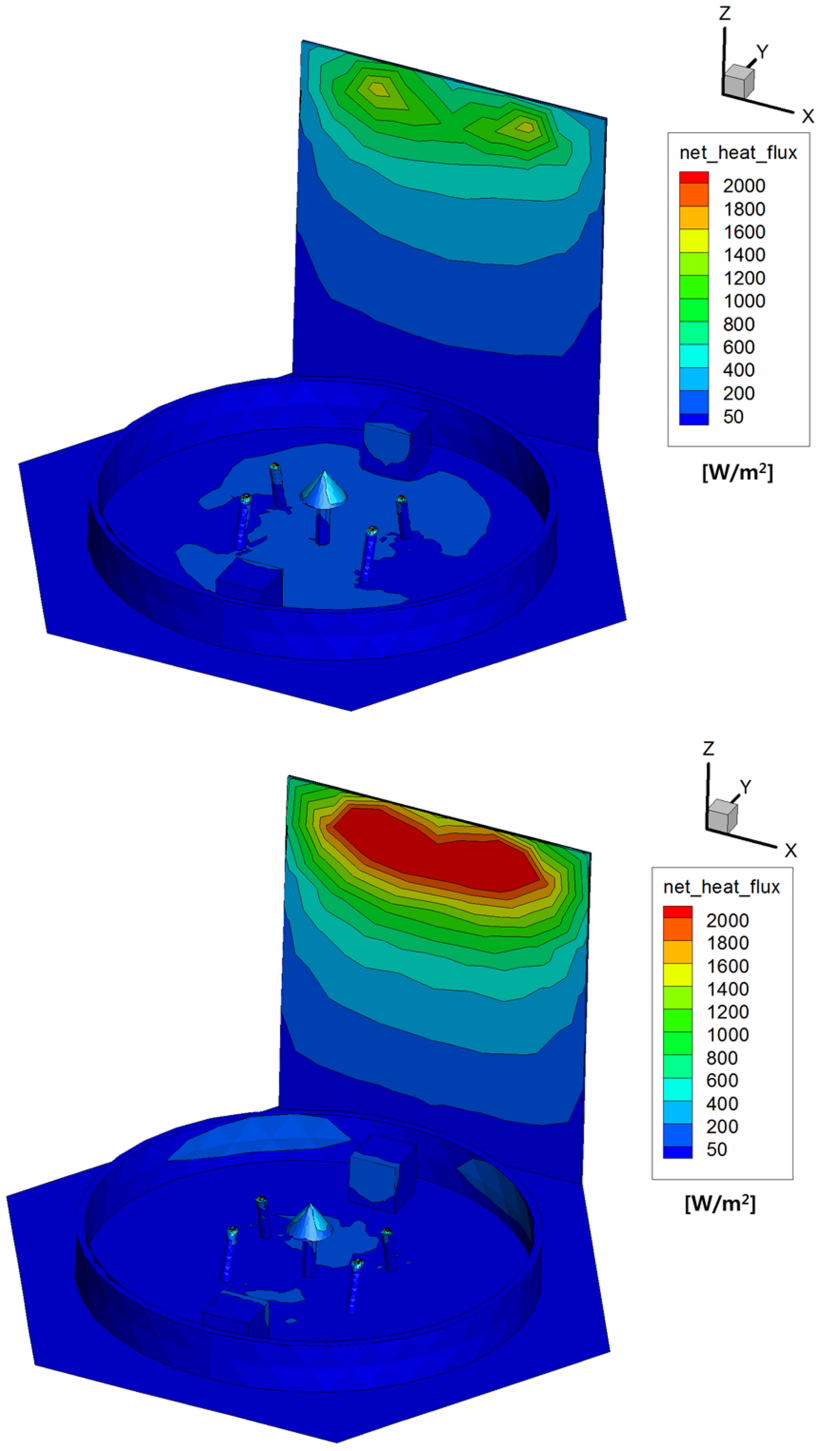
Surface heat flux distributions of plume gas flow [W/m^2^]. (A) Monopropellant hydrazine. (B) Bipropellant MMH-NTO.

As a second outcome, distributions of the number flux and heat flux of the two plume gases on the satellite components and structure were investigated to compare the plume impingement influences. Fig 10 clearly shows that a highly intensive gas particle distribution greater than 1E+22 1/m^2^·s was found on the upper regions of the solar panel and the S-band antenna because they were directly exposed to the plume flow. Especially, a larger number of the hydrazine plume gas particles were extensively distributed over the surfaces of the antenna components inside the interface ring and the solar panel compared to that of the MMH-NTO gas because a more dense combustion gas of the hydrazine propellant was exhausted from the thruster and collided onto the satellite as predicted from the streamline and density behaviors of the plume particles. Additionally, the surface heat flux on the solar panel was similar to the number flux result shown in Fig 11 because its distribution is directly proportional to the number of plume particles impinged on the surfaces of the satellite. However, an intense heat flux over 2,000 W/m^2^ was generated by the MMH-NTO plume gas on the upper area of the solar panel whereas the maximum value of the hydrazine gas was predicted to be less than about 1,500 W/m^2^ which was not relatively high compared with the solar constant of *q*_sol_ = 1353 W/m^2^ [21]. Therefore, it was anticipated that a more excessive thermal loading can be transferred to the spacecraft if the MMH-NTO thruster is used because of the higher chemical energy inherent in the bipropellant itself which is similar to the overall temperature results of the plume particles.

**Fig 12:**
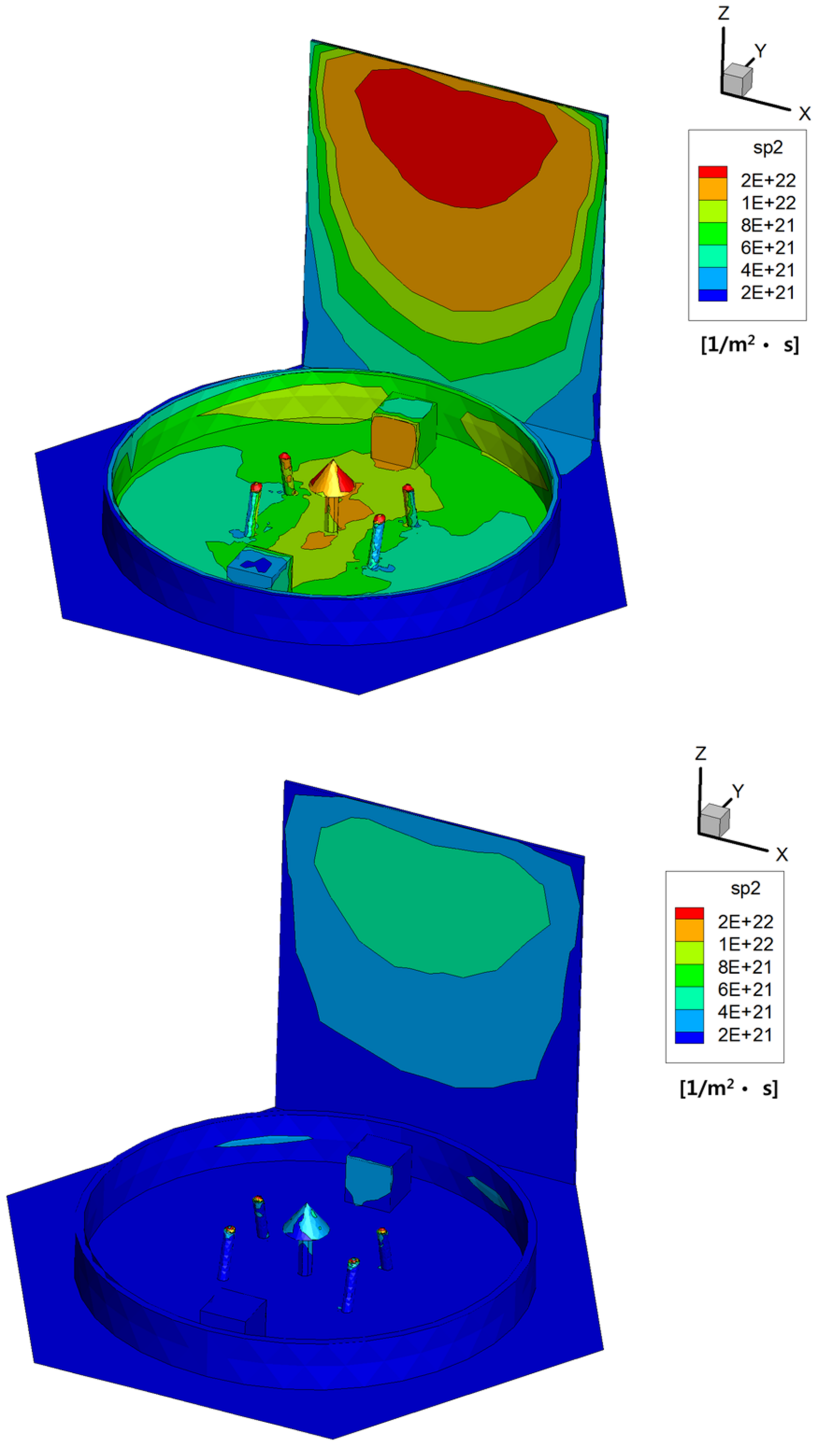
Surface number flux distributions of *H*_*2*_ species [1/m^2^·s]. (A) Monopropellant hydrazine. (B) Bipropellant MMH-NTO.

To consider the influence of the plume backflow in more detail, surface distributions of *H*_*2*_ species were investigated for both the propellants shown in Fig 12. Because it has the lightest molecular weight among the various gas compositions, previous studies revealed that *H*_*2*_ separated strongly from the main flow stream compared to other gas species, and thus, it was a main gas ingredient of the hydrazine plume backflow [10,12]. In the case of the hydrazine propellant, Fig 12A shows that *H*_*2*_ species was distributed more spaciously over not only the solar panel but also over the bottom platform region of the satellite inside the interface ring compared to the MMH-NTO propellant in Fig 12B because a larger mole numbers of *H*_*2*_ was produced initially from the chemical reaction process of the hydrazine propellant inside the thruster chamber as given in Table 1. Accordingly, this chemical separation of *H*_*2*_ with the higher mole fraction contained in the hydrazine plume backflow also transferred the local heat flux over a more extensive area inside the interface ring compared to the MMH-NTO propellant from Fig 11B. As a consequence, it is predicted that the higher collisions of the hydrazine plume particles can cause more critical impacts on the spacecraft in terms of a disturbance force/torque and a contamination of the chemical species because a more dense decomposition gas is produced from the monopropellant hydrazine propellant. Moreover, the components and structures located adjacent to the hydrazine thruster will be influenced greatly by the *H*_*2*_ molecules because a relatively larger amount of *H*_*2*_ is separated from the main plume stream and included in the backflow as a major species. Thus, surface contamination by the deposition of the plume particles including a considerable amount of *H*_*2*_ onto the spacecraft could be a significant problem for sensitive equipment such as solar cells, optical equipment and GaAs microwave devices when the monopropellant hydrazine thruster is used.

**Fig 13:**
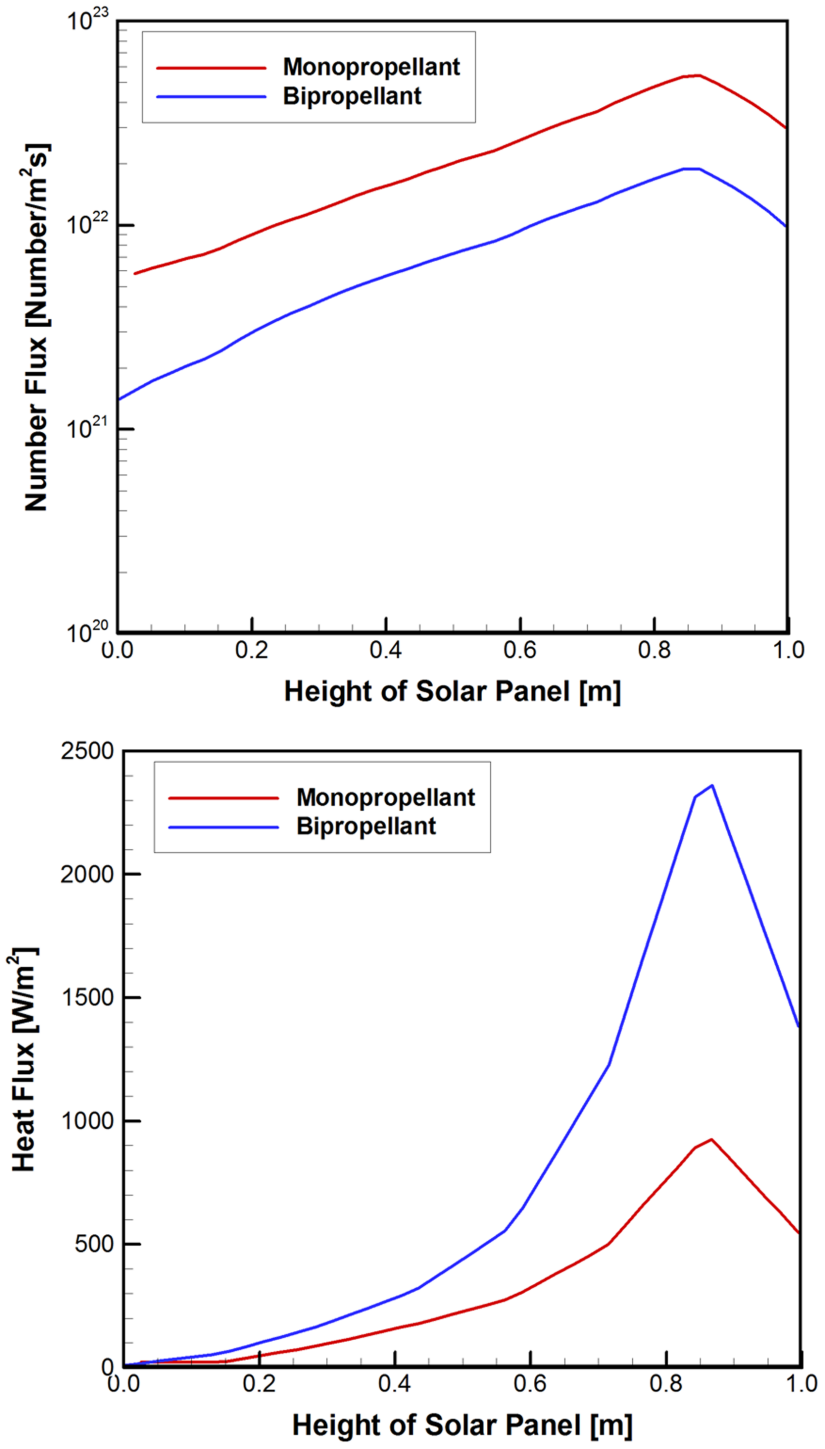
Surface flux distributions of the plume gas flow at the center of solar panel in *z*-axis. (A) Surface number flux [1/m^2^·s]. (B) Surface heat flux [W/m^2^].

For a quantitative comparison, the number flux and the surface heat flux variations of the plume gas were measured at the center of the solar panel following the *z*-axis for both the propellants and plotted in Fig 13. Similar to the previously examined number density and overall temperature results, these surface flux variations of the two plume gases also increased gradually from the bottom to the top of the panel, while a higher level of the number flux was maintained for the hydrazine propellant and vice versa for the surface heat flux.

As a final result, the disturbance forces and torques induced by the thruster plume impingement was evaluated in Table 3 as relative values to absolute nominal ones. From the previous plume streamline and surface number flux distributions of the two propellants, the larger disturbance force and torque were predicted to be caused by the hydrazine plume impingement rather than by the MMH-NTO. From Table 3, it could be inferred that the collision between the plume backflow and the satellite structure was more dominant for the hydrazine propellant because the disturbance forces of the hydrazine propellant were greater in the *x* and *y* axes due to the considerable amount of exhaust plume particles in the backflow regime with the separation of *H*_*2*_. However, a main stream of the MMT-NTO plume gas with a lower density tended to flow straight following the axial direction of the nozzle rather than diffuse in the radial direction, which caused less plume backflow impingement on the satellite. Additionally, a larger number of the hydrazine plume particles induced a much more severe level of disturbance torques in the *x* and *y* axes than that of the MMT-NTO gas because of a direct plume impingement effect on the solar panel.

**Table 3:** Predictions of relative disturbance force and torque values of two plume gases.

Disturbance Force	*x*	*y*	*z*
Absolute Nominal Value [N]	0.000	0.000	19.404
Monopropellant Hydrazine [%]	0.103	42.753	0.269
Bipropellant MMH-NTO [%]	0.010	35.549	1.248
Disturbance Torque	*x*	*y*	*z*
Absolute Nominal Value [N· m]	0.087	0.034	0.000
Monopropellant Hydrazine [%]	1079.057	17.416	0.974
Bipropellant MMH-NTO [%]	756.457	0.751	0.081

## Conclusions

The present study conducted numerical analysis to investigate and compare major differences of the plume gas flow impingement effects quantitatively between the small mono- and bipropellant thrusters. To increase an efficiency of the numerical calculations, the computational fluid dynamics (CFD) methods with the N–S equations and the parallelized DSMC method were employed for the different calculation flow regimes depending on the flow conditions. Major differences of the plume gas impingement effects between the two propellants were summarized from the present results.

1. The monopropellant hydrazine plume gas flow spread more densely all over the calculation domain including the backflow region than that of the MMH-NTO gas because a combustion gas with a higher density was produced inside the chamber. Consequently, the higher collisions of the hydrazine plume particles caused more critical impacts for the spacecraft in terms of a disturbance force/torque and a contamination of the chemical species compared to the MMH-NTO propellant.
2. Components and structures located adjacent to the hydrazine thruster was influenced greatly by the *H*_*2*_ molecules because a relatively larger amount of *H*_*2*_ was separated from the main plume stream and was included in the backflow as a major species. Thus, surface contamination by the plume molecule deposition including a considerable amount of *H*_*2*_ onto the spacecraft could be a significant problem for the any *H*_*2*_ sensitive equipment when the monopropellant hydrazine thruster is used.
3. A much more excessive heat flux was transferred to the spacecraft when the MMH-NTO thruster was used because of a higher chemical energy inherent in the bipropellant itself. As the plume gas temperature was greatly dependent on the amount of thermal energy released from the chemical reactions of the propellant, it was anticipated that plume gas impingement could exert a severe thermal loading influence on the spacecraft components and structure depending on the type of propellant used.

From the present analysis results, it could be found that the impingement influences of the plume gas on the spacecraft components and structure were highly correlated with the chemical reaction characteristics of the propellant used. Thus, if small mono- and/or bipropellant thrusters are to be used, then a careful understanding of not only the performance characteristics of these two propellants but also the impinging effects of their plume gases is required to design the spacecraft efficiently. Consequently, it is anticipated that the present study could provide practically useful data to the related engineers on the optimized design philosophy of the spacecraft and the selection of the proper propulsion system through detailed investigations of the plume impingement effects of the mono- and bipropellant thrusters, which can finally lead to reduction of development costs and time from the initial design phase.

## Abbreviations

$d\vec{v_{1}}$: molecules of class with velocity *v*_1_
*dΩ*: elementary solid angle
$\vec{F}$: external force vector
*f*: probability density function of molecules
*M*: molecular mass [g/mol]
*m*: mass [kg]
*n*: number density [1/m^3^]
*p*: pressure [N/m^2^]
*R*: gas constant [8314 J/kmol K]
*r*: radial direction distance [m]
$\vec{r}$: position vector of molecules
*T*: temperature [K]
*t*: time [sec]
$\vec{v}$: velocity vector of molecules
*v_r_*: relative velocity of molecules
*x*: *x* direction distance [m]
*Y*: mass fraction
*y*: *y* direction distance [m]
*z*: *z* direction distance [m]
*ρ*: density [kg/m^3^]
*σ*: collision cross section
*: post-collision value
*i*: *i*^*th*^ chemical species
